# Genome-Wide Selection Signals Reveal Candidate Genes Associated with Plateau Adaptation in Tibetan Sheep

**DOI:** 10.3390/ani14223212

**Published:** 2024-11-08

**Authors:** Yufang Song, Chao Yuan, Xuejiao An, Tingting Guo, Wentao Zhang, Zengkui Lu, Jianbin Liu

**Affiliations:** 1 Key Laboratory of Animal Genetics and Breeding on the Tibetan Plateau, Ministry of Agriculture and Rural Affairs, Lanzhou Institute of Husbandry and Pharmaceutical Sciences, Chinese Academy of Agricultural Sciences, Lanzhou 730050, China; songyf805@163.com (Y.S.); yuanchao@caas.cn (C.Y.); anxuejiao@caas.cn (X.A.); guotingting@caas.cn (T.G.); m18251871965@163.com (W.Z.); 2 Sheep Breeding Engineering Technology Research Center, Chinese Academy of Agricultural Sciences, Lanzhou 730050, China

**Keywords:** high-altitude adaptation, Tibetan sheep, *F*_ST_, θπ ratio, selection signal

## Abstract

Tibetan sheep have developed unique adaptations for survival in the Qinghai–Tibet Plateau environment. However, the functional genes and molecular mechanisms that regulate hypoxia adaptation have not been fully characterized. In this study, based on the whole-genome resequencing data for Tibetan sheep at different altitudes, the population differentiation index (*F*_ST_) and nucleotide diversity ratio (θπ ratio) were evaluated in populations of 20 Oula sheep (3501 m altitude, OL), 20 Zashijia sheep (4369 m altitude, ZSJ), and 20 Awang sheep (4643 m altitude, AW) to reveal candidate loci related to high-altitude hypoxia. We screened 728 and 524 candidate genes in the AW vs. OL and ZSJ vs. OL groups, respectively, of which 134 genes were jointly screened. Candidate genes were mainly enriched in the Ras, melanoma, melanogenesis, VEGF, and PPAR signaling pathways. *HIF1AN*, *PDGFA*, *PDGFD*, *ANXA2*, *SOCS2*, *NOXA1*, *WNT7B*, *MMP14*, *GNG2*, *ATF6*, *PGAM2*, *PPP3R1*, *GSTCD*, and *PPARA* may play important roles in the high-altitude adaptation of Tibetan sheep. In conclusion, this study provides valuable insights into the genes and molecular mechanisms that underlie high-altitude hypoxia adaptation in Tibetan sheep.

## Simple Summary

Understanding how animals adapt to extreme environments, such as high altitudes, is vital for conservation efforts and animal welfare. Tibetan sheep residing on the Qinghai–Tibet Plateau possess unique adaptive traits, enabling them to survive in the severe conditions of high altitudes and low oxygen. However, the specific genes and molecular mechanisms that contribute to this adaptation in Tibetan sheep remain largely unidentified. So, based on whole-genome resequencing data for Tibetan sheep at different altitudes, this study analyzed the population structure and genomic differences of three Tibetan sheep breeds through *F*_ST_ and θπ ratio methods. In addition, we identified a series of candidate genes that may be related to the high-altitude adaptation of Tibetan sheep. This study provides a theoretical basis for the molecular mechanism of adaptation to extreme environments in Tibetan sheep and lays down a cornerstone for research in related fields.

## 1. Introduction

Tibetan sheep are one of the three prominent coarse-wooled sheep breeds in China, inhabiting the Tibetan Plateau at an altitude of 2000 to 5000 m. This breed is an important germplasm resource on the Tibetan Plateau and is extremely adaptable to the challenging environmental conditions of the plateau, including resistance to low temperatures, low pressures, and intense ultraviolet radiation [1]. Owing to the unique climatic characteristics, the Tibetan Plateau exerts significant pressure on local organisms and has created a large number of endemic species with various physiological, biochemical, and morphological genetic characteristics that are stable in its low-oxygen environment [2]. With recent advances in biotechnology, research has increasingly focused on mechanisms underlying plateau adaptation in different species. Using whole-genome sequencing technology, researchers have successfully detected selection signals related to high-altitude adaptations in various taxa such as humans [3,4], yaks [5], and pigs [6]. These findings deepen our understanding of the evolutionary processes of organisms surviving in extreme environments and provide an important reference for human plateau disease research.

Tibetan sheep are one of the major livestock breeds in the Tibetan Plateau region of China and are the main source of production and living resources for herders, with a large and widely distributed population [7]. Tibetan sheep are well adapted to the ecological conditions of the alpine pasture and rough feeding, exhibiting stable genetic characteristics, strong resistance to adverse conditions, great meat quality, and excellent carpet wool quality [8,9,10]. Meanwhile, in the long-term evolution of low-oxygen tolerance, Tibetan sheep have formed unique morphological, physiological, ecological, and genetic characteristics [11]. However, there are major technical bottlenecks in the Tibetan sheep industry, such as low production performance, insufficient exploitation of genetic potential, and inefficient breeding technology, that seriously constrain breeding and the development of high-quality, healthy, and sustainable populations. Tibetan sheep are native to the Tibetan Plateau and are mainly distributed in Tibet, Qinghai, Gansu, Sichuan, Yunnan, and Guizhou [12], with variation breeds corresponding to differences in ecological conditions across the region. Oula sheep (OL), Zashijia sheep (ZSJ), and Awang sheep (AW) are well adapted to the low pressure, cold temperatures, and humidity in the alpine grasslands and to four-season grazing and year-round, multi-camp grazing management. With a typical eco-geographical distribution, they have become excellent breeds adapted to the plateau environment. The specificity and complexity of the ecological conditions in different regions have resulted in numerous Tibetan sheep varieties with differences in ecological adaptability [13]. Therefore, these animals have become ideal models for studying the mechanism underlying adaptation to hypoxia in plateau animals.

The rapid development of high-throughput sequencing technology has provided powerful tools for unraveling livestock domestication history [14], environmental adaptation mechanisms [15], and traits under selection [16]. Genome-wide selection signatures have been extensively applied in studies of plant and animal evolution [17]. Guo et al. [18] conducted selection signal analysis on six Tibetan goat breeds and found that the *EPAS1* gene was associated with the goats’ high-altitude adaptations. *CDK2*, *SOCS2*, *NOXA1*, and *ENPEP* contribute to Qinghai–Tibetan goats’ adaptations to harsh high-altitude environments [19]. Genes related to hypoxia were also found in yaks (*ADAM17*, *ARG2*, and *MMP3*) [5] and Tibetan antelopes (*ADORA2A*, *CCL2*, *ENG*, *PIK3C2A*, *PKLR*, *ATP12A*, and *NOS3*) [20]. Furthermore, Wei et al. [21] conducted whole-genome resequencing of seven Tibetan sheep breeds and screened a series of candidate genes associated with adaptation to the hypoxic plateau environment, including *EPAS1*, *PPARG*, *CRYAA*, *LONP1*, *SOCS2*, *DPP4*, *SOD1*, and *NF1*. They demonstrated that *EPAS1* played a central role in the regulatory network involved in the adaptation to high-altitude hypoxia. The above results suggest that there may be differences in the genes selected between species, despite similar environmental conditions, and that different species may possess different high-altitude adaptation mechanisms.

Tibetan sheep are the most abundant domestic animal on the Tibetan Plateau, living in a high-altitude, low-oxygen environment for a long period, forming a unique genetic adaptation mechanism. Understanding the genetic adaptation mechanisms of Tibetan sheep can reveal the uniqueness and importance of their genetic resources and also provide a scientific basis for the development of animal husbandry on the plateau, promoting the sustainable utilization of animals’ genetic resources. Whole-genome sequences of Tibetan sheep raised at different altitudes were the subject of this investigation. Signatures of selection were localized using the population differentiation index (*F_ST_*) and nucleotide diversity ratio (θπ ratio). Our results revealed candidate genes related to altitude acclimatization that can provide a reference for further research aimed at the in-depth excavation of genes related to high-altitude hypoxia acclimatization in Tibetan sheep.

## 2. Materials and Methods

### 2.1. Sample Collection and Resequencing

All experimental studies involving sheep were approved by the Animal Ethics Committee at the Lanzhou Institute of Husbandry and Pharmaceutical Sciences, Chinese Academy of Agricultural Sciences (NO. 20231447). Blood samples were collected from 60 individuals from three Tibetan sheep breeds (Table 1), including 20 OL, 20 ZSJ, and 20 AW. All experimental sheep were randomly selected, and their whole blood samples were collected using the jugular vein method and stored at −20 °C for future use. Genomic DNA was extracted from blood samples using a blood genome extraction kit (Tiangen Biotech Co., Ltd., Beijing, China). The concentration and purity of DNA were detected using a NanoDrop 2000 nucleic acid protein analyzer (Thermo Fisher Scientific, Wilmington, NC, USA). DNA from 60 sheep were genotyped on the Illumina HiSeq X10 PE150 platform.

### 2.2. Quality Control and Reference Genome Alignment

Before assembly and data analyses, the raw reads were filtered, and the results were stored in FASTQ file format. Next, stricter filtering was applied to obtain high-quality clean reads for subsequent analyses. The filtering steps were as follows: adapters were removed, reads containing a proportion of unknown nucleotides (N) greater than 10% were removed, and the low-quality reads (in which bases with a quality value of Q ≤ 20 accounted for more than 50% of the full-length read) were removed.

The Huoba Tibetan Sheep reference genome assembled by our team was selected for comparison. The filtered reads were compared using the mem algorithm of BWA (0.7.15) [22] (parameters: -k 32-M). The results were exported to a BAM format file using SAMtools (v 1.17) [23], and then duplicate reads were labeled using Picard (2.18.7) (http://sourceforge.net/projects/picard/, (accessed on 1 June 2024)) software. The sequencing depth and coverage were determined using BEDTools statistics [24].

To improve the accuracy of the data analyses, PLINK 1.09 software [25] was used for quality control to remove unqualified single nucleotide polymorphisms (SNPs), with the following quality control criteria: (1) SNP detection rate greater than 95%; (2) *p* > 0.0001 in the Hardy–Weinberg equilibrium test; (3) SNPs with a minimum allele frequency (MAF) greater than 0.05; (4) unlocalized SNPs were removed and loci on autosomes were selected for subsequent analyses.

### 2.3. Population Structure Analysis

To understand the clustering patterns and genetic correlations among the three populations, a principal component analysis (PCA) was performed using PLINK 1.09 [25] software. Additionally, to assess the genetic relatedness among individuals, we constructed neighbor-joining (N-J) trees using TreeBeST software (https://github.com/Ensembl/treebest, accessed on 6 June 2024) [26] and visualized them using iTOL (v6) [27] software (https://itol.embl.de/upload.cgi, (accessed on 6 June 2024)). In addition, to further confirm the results of the PCA and N-J trees, this study used admixture (v 1.3) software [28] for population structure analysis, with K values set from 2 to 4 to construct the population genetic structure, and the cross-validation (CV) error to determine the optimal K.

### 2.4. Selection Signal Analyses

This study used *F*_ST_ and θπ ratios to detect the degree of genetic differentiation between different populations. These values were calculated using VCFtools (0.1.15) [29]. *F*_ST_ is widely used to identify genome-wide signals of selection based on SNPs [30]. *F*_ST_ values are calculated based on differences in allele frequencies between populations and range from 0 to 1 [30], with larger values indicating a higher degree of differentiation and 0 indicating that there are no differentiated loci in the population [31]. The θπ represents nucleotide diversity, and the θπ ratio is the ratio between two populations based on gene heterozygosity. As the degree of selection increases, polymorphism decreases. Using filtered SNPs and PopGenome software [32], we analyzed nucleotide diversity within the population and compared diversity between populations using a sliding window method. The *F*_ST_ and θπ ratio methods were used to jointly screen for strong selection signals for the target genes and provide more comprehensive and accurate genetic information. Loci within the windows ranked in the top 5%, based on *F*_ST_ and θπ ratios, were screened as significant SNPs and candidate loci under selection. Genomic annotation of the candidate loci was performed using ANNOVAR software (https://annovar.openbioinformatics.org/en/latest/, (accessed on 10 June 2024)) [33].

### 2.5. Candidate Gene Enrichment Analysis

DAVID 6.8 (https://david.ncifcrf.gov/, (accessed on 15 June 2024)) and Kobas 3.0 (http://bioinfo.org/kobas, (accessed on 15 June 2024)) were used for a gene ontology (GO) functional enrichment analysis and a Kyoto Encyclopedia of Genes and Genomes (KEGG) pathway enrichment analysis of annotated functional genes. *Ovis_aries* was chosen as the background organism, and *p* < 0.05 was used as the significance threshold. The terms and pathways were further searched using the NCBI database (https://www.ncbi.nlm.nih.gov/, (accessed on 11 July 2024)) to screen for functional genes related to altitude traits in Tibetan sheep.

## 3. Results

### 3.1. Genetic Variation and Population Genetic Analysis

**Figure 1:**
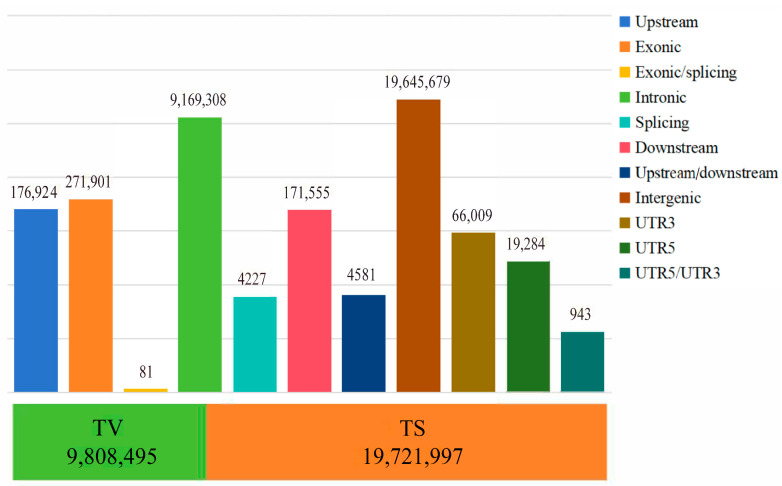
The distribution of SNP variants in genome regions. The bar chart above indicates location information; the stacked graph below represents conversion and inversion information.

In this study, the average sequencing depth for 60 Tibetan sheep was 6.1×, and a total of 109,644,260 high-quality reads (15,774,610,264 bp) were obtained after quality control. A total of 29,530,492 SNPs were obtained after comparison with the reference genome. Genome annotation (Figure 1) revealed that 19,645,679 SNPs were located in intergenic regions, 9,169,308 SNPs were located in intronic regions, and 271,901 SNPs were located in exons. The TS/TV (transition/transversion) ratio was 2.01, indicating that the genome was in equilibrium and relatively structurally conserved. AW, ZSJ, and OL had 2,348,044, 2,399,424, and 2,435,327 SNPs, respectively, including 2,249,434 SNPs shared by AW and OL and 2,295,302 SNPs shared by ZSJ and OL. The high percentage of shared SNPs suggests that the populations share a high degree of similarity at the genetic level and also implies that the populations have experienced some common adaptive traits, which may be related to environmental adaptations.

**Figure 2:**
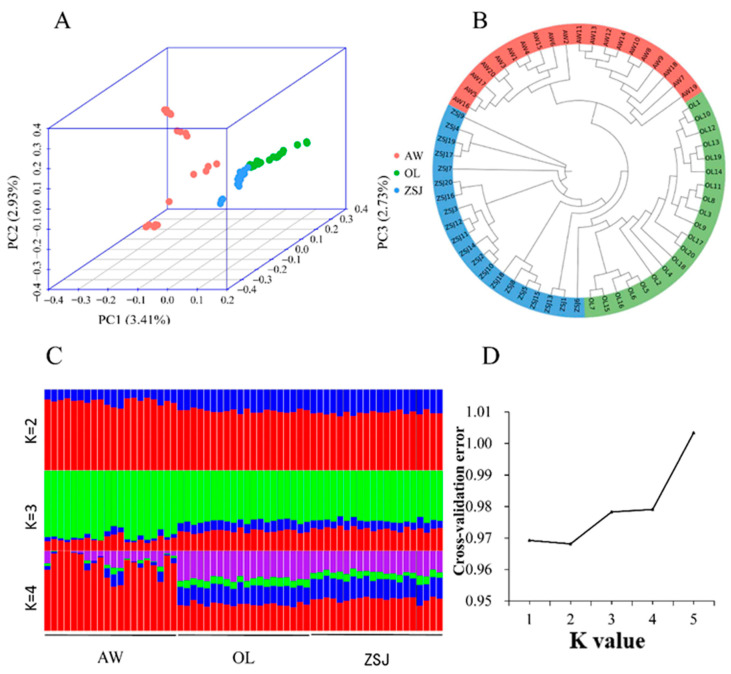
Analysis of the population genetic structure. (**A**) Principal component analysis (PCA); (**B**) phylogenetic tree (generated using the neighbor-joining method); (**C**) population structure analysis (K = 2, 3, or 4, Different colors represent different components of ancestry); (**D**) cross-validation error.

After quality control, the genotype data were analyzed via a principal component analysis, phylogenetic tree construction, and an analysis of population structure. In the PCA (Figure 2A), PC1 explained 3.41% of the genetic variation, clearly separating AW from the other populations, and PC2 (2.93% genetic variation) results showed some crossover between the three populations. PC3 (2.73% genetic variation) separated OL from the three populations. Because the proportion of variation explained by PC1, PC2, and PC3 is low and some crossover exists between the three groups, these three groups are the same population. Based on the N-J tree (Figure 2B) results, the three populations were clearly clustered into three categories. Population structure analysis found that the CV error was the smallest when K = 2 (Figure 2C,D), and there was no significant difference between the three populations. When K = 3 or K = 4, the background compositions of ZSJ and OL groups became more similar, and this result was consistent with the PCA results. Combined with population structure analysis and CV error, the ancestors of these three populations should be the same population.

### 3.2. Analysis of Selection Signals

**Figure 3:**
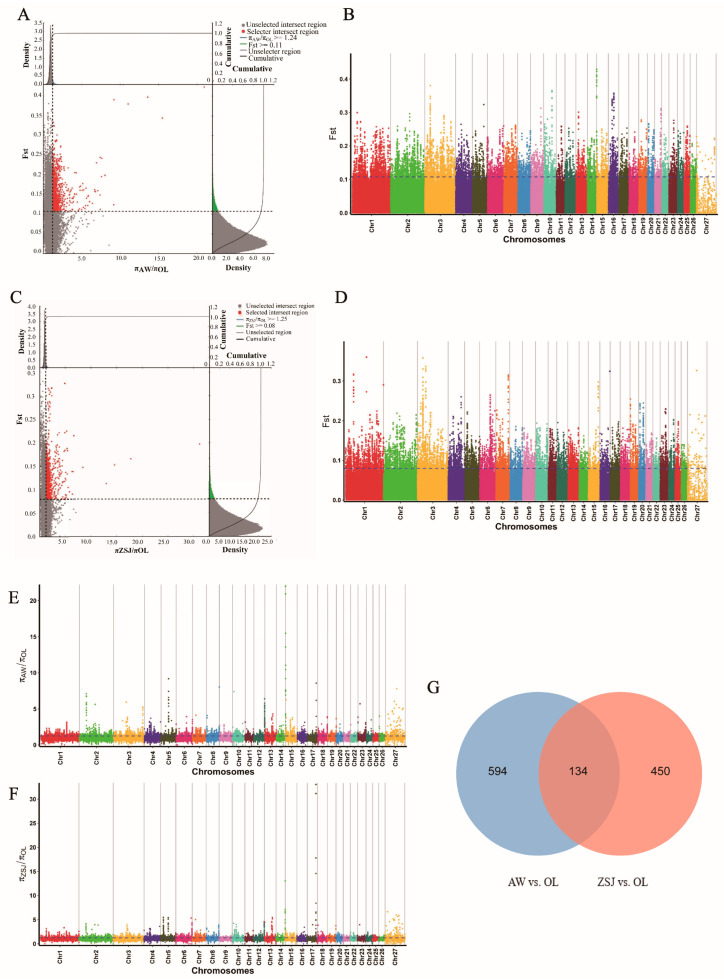
Analysis of selection signals. (**A**) *F*_ST_ and θπ ratio joint selection elimination (AW vs. OL); (**B**) genome-wide distribution of *F*_ST_ (AW vs. OL); (**C**) *F*_ST_ and θπ ratio joint selection elimination (ZSJ vs. OL); (**D**) genome-wide distribution of *F*_ST_ (ZSJ vs. OL); (**E**) genome-wide distribution of θπ ratios (AW vs. OL); (**F**) genome-wide distribution of θπ ratios (ZSJ vs. OL); (**G**) Venn diagram of overlapping genes in the two comparisons.

SNP loci were jointly screened using the *F*_ST_ and θπ ratio methods, setting the top 5% as the screening criterion. In total, 2903 loci were screened in the AW vs. OL group (Figure 3A,B,E), and 728 candidate genes were annotated. A total of 2654 loci were screened in the ZSJ vs. OL (Figure 3C,D,F) group, and 524 candidate genes were annotated. Two comparison groups jointly screened 134 overlapping candidate genes (Figure 3G), among which *HIF1AN*, *PDGFA*, *PDGFD*, *ANXA2*, *SOCS2*, *NOXA1*, *WNT7B*, *MMP14*, *GNG2*, *ATF6*, *PGAM2*, *PPP3R1*, *GSTCD*, and *PPARA* may be related to high-altitude adaptation.

### 3.3. Enrichment Analysis of Candidate Genes

**Figure 4:**
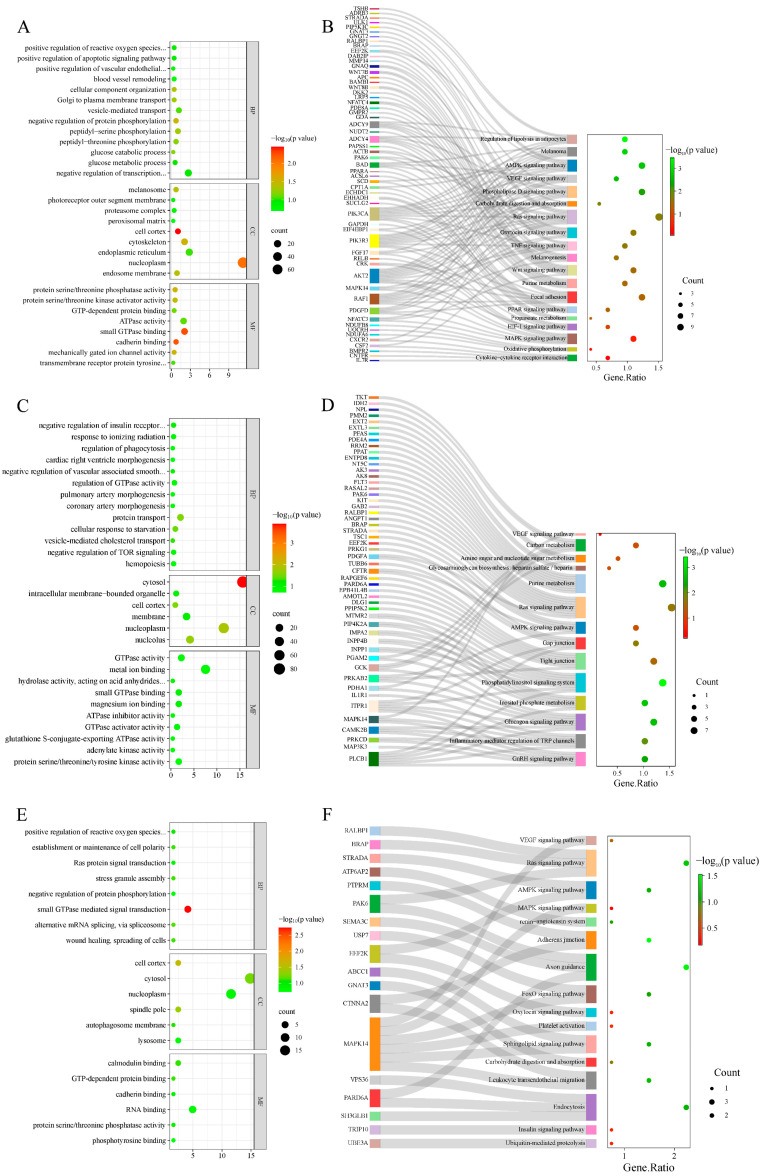
GO enrichment and the KEGG pathway enrichment results. (**A**) GO terms enriched in AW vs. OL; (**B**) AW vs. OL enriched the KEGG pathway; (**C**) GO terms enriched in ZSJ vs. OL; (**D**) ZSJ vs. OL enriched KEGG pathway; (**E**) GO terms enriched with overlapping genes in two groups; (**F**) the KEGG pathway enriched with overlapping genes in two groups.

GO enrichment analysis was performed on selected genes from the AW vs. OL group (Figure 4A, Table S1), and a total of 136 GO terms were obtained, with 26 significant terms (*p* < 0.05). A total of 111 GO terms were obtained from the ZSJ vs. OL group (Figure 4C, Table S2), including 21 significant GO terms (*p* < 0.05). Furthermore, GO enrichment analysis of the genes shared between the two comparisons revealed four significant GO terms (Figure 4E, Table S3) (*p* < 0.05). The GO terms associated with high-altitude adaptation included the positive regulation of reactive oxygen species metabolic processes, the vascular endothelial growth factor signaling pathway, blood vessel removal, and melanosomes and the negative regulation of vascular associated smooth muscle cell migration and hemopoiesis.

In the AW vs. OL group (Figure 4B, Table S4), the genes were significantly enriched in 94 signaling pathways, including seven pathways associated with altitude acclimatization (the AMPK, Ras, VEGF, melanogenesis, purine metabolism, melanoma, and PPAR signaling pathways). In the ZSJ vs. OL group (Figure 4D, Table S5), four enriched pathways related to altitude were identified (the AMPK, Ras, purine metabolism, and VEGF signaling pathways). KEGG enrichment analysis of the genes shared between the two groups (Figure 4F, Table S6) revealed 92 pathways, including four pathways related to altitudinal adaptation (the AMPK, Ras, and VEGF signaling pathways, and the renin–angiotensin system).

## 4. Discussion

During the long evolutionary process, animals at high altitudes underwent irreversible and heritable adaptive structural changes in their cardiovascular systems, as well as in other tissues and organs, enabling survival and reproduction in low-oxygen environments [34]. Qiu et al. [5] sequenced the genome and transcriptome of female yaks and found that genes related to energy metabolism and hypoxia adaptation in yaks underwent positive selection, which further highlighted the unique adaptability of yaks to extreme environments. In addition, Ge et al. [20] used genome construction technology to pinpoint genes related to repair mechanisms, angiogenesis, and hypoxia tolerance in Tibetan antelopes. These genes underwent positive selection, which conferred Tibetan antelopes with a remarkable ability to survive in the highland environment. Thus, in the field of animal husbandry, yaks, Tibetan pigs, and Tibetan sheep that have lived in high-altitude environments for generations have become ideal models for studying the mechanisms underlying low-oxygen adaptations. In this study, Tibetan sheep at different altitudes were selected for whole-genome resequencing to analyze the genomic signals of selection associated with high-altitude adaptation. The TS/TV ratio is commonly used to measure the quality of SNPs and can also reflect the proportions of homozygous and heterozygous SNPs in a species. The TS/TV ratio was 2.01, which is close to the theoretical value of 2 [35], indicating that the genome is in equilibrium and is relatively structurally conserved. These results show that the random sequencing error was low, confirming the reliability of the sequencing data.

In this study, the population genetic structure and CV analyses of the three Tibetan sheep groups showed that their ancestors should be from the same population and that, despite differences in geographical distribution, similar genetic characteristics and adaptive mechanisms were formed among them, probably due to long-term natural selection and environmental adaptation. This similarity was visualized in the PCA plots, which showed the overlapping and mixing of individuals of different strains in the genetic space. This finding was consistent with the phylogenetic analysis and geographical locations of the populations. The *F*_ST_ and θπ ratios were used to screen for significant SNPs in high-altitude Tibetan sheep breeds [35], followed by functional enrichment analyses of candidate genes. The combined analysis of *F*_ST_ and θπ ratios for screening and identification can effectively improve the reliability and accuracy of selection signals [36,37].

Plateau animals have evolved unique adaptations to survive in low-oxygen environments. Tibetan sheep are well-adapted to the ecological environment of alpine grasslands, and this adaptation may be determined by the synergistic effects of multiple genes [38]. Based on the functional enrichment analysis conducted in this study and in previous literature, the majority of genes involved in hypoxia adaptation were associated with the HIF (hypoxia-inducible factor) pathway [38]. Under hypoxic conditions, the Ras signaling pathway functions by interacting with the HIF pathway to promote angiogenesis [39]. A study on Tibetan goats [13] found that most genes related to low-oxygen adaptation were enriched in the melanoma and VEGF signaling pathways. Similar findings have been reported in Tibetan sheep [21] and Tibetan pigs [40]. In a study of Tibetan goats [13], most genes related to low-oxygen adaptations were enriched in the melanoma and VEGF signaling pathways, and similar reports have been found in Tibetan sheep [21] and Tibetan pigs [40]. Furthermore, research has demonstrated that high-altitude hypoxic environments significantly impact animals’ fat metabolism [41,42,43]. PPAR is a nuclear receptor that regulates the transport, esterification, and oxidation of fatty acids, playing a critical role in maintaining cellular energy balance and preventing lipid peroxidation. In addition, it has been shown that the PPAR signaling pathway plays an important role in regulating various physiological processes, including adipocyte differentiation, lipid metabolism, and glucose metabolism [44,45].

Plateau adaptation is complex and involves multiple factors, such as hypoxia-inducible factors (HIFs), angiogenesis, vasodilation, and glycolytic metabolism [15]. Among these, HIFs are a class of transcription factors that play a central role in cellular perception and adaptation to changes in oxygen levels, consisting of α subunits (HIF-1α, HIF-2α, and HIF-3α) and β subunits (HIF-1β) [46]. In low-oxygen environments, *HIF1AN* (hypoxia-inducible factor-1 subunit alpha inhibitor) hydroxylates Asn803 at the carboxyl end of the HIF-1α subunit, blocking the interaction between HIF-1α and the transcriptional cofactor P300 [47]. This inhibits the transcriptional activity of HIF-1α while maintaining its stability. Additionally, *HIF1AN* is associated with the hypoxic response in ground tits (*Parus humilis*) [48]. Therefore, it has been speculated that *HIF1AN* may also play a crucial role in the adaptation of Tibetan sheep to high-altitude and low-oxygen environments. Zhang et al. found that the VEGF signaling pathway plays an important role in the adaptation of the myocardium to hypoxia in Tibetan pigs [40]. In this study, *MAPK14* in the VEGF signaling pathway was identified, suggesting that this gene is associated with the response to hypoxia in Tibetan sheep. PDGFs (platelet-derived growth factors) are cytokines released by platelets and include four subtypes (*PDGFA*, *PDGFB*, *PDGFC*, and *PDGFD*). The HIF pathway is essential for the response to hypoxia in postnatal animals [49]. In the present study, *PDGFD* was involved in the Ras signaling pathway, which plays an important role under hypoxic conditions by interacting with the HIF pathway to promote angiogenesis. *ANXA2* (Annexin A2) is an important member of the membrane-associated protein family and a calcium-dependent phospholipid binding protein. In Tibetan pigs, the *ANXA2* gene might be related to adaptation to hypoxic conditions [40]. *ANXA2* may regulate physiological responses under hypoxic conditions by affecting cellular signaling pathways. *NOXA1* (NADPH oxidase activator 1) is an activator of NOX1, which is associated with the response of HIF-1 to intermittent hypoxia [50]. In addition, *SOCS2* (suppressor of cytokine signaling 2) has been identified as a candidate gene for adaptation to hypoxia, and it is related to the role of HIFs in promoting the *EPO* (erythropoietin) response to hypoxic conditions [51]. In a study of Tibetan goats [21], *SOCS2* was identified as a candidate gene related to high-altitude hypoxia, similar to the results of this study. On the basis of resequencing data, Wang et al. [19] also found that *SOCS2* and *NOXA1* play important roles in the adaptation to hypoxia in Tibetan goats in Qinghai.

*WNT7B* (wingless-type MMTV integration site family member 7B) regulates angiogenesis in the lungs and heart and serves as an important mediator for hypoxia-induced angiogenesis [52]. MMPs (matrix metalloproteinases) are zinc-dependent endopeptidases with roles in various physiological processes. *MMP14* is an important member of the MMP family and the only protease in the family that promotes cell migration in collagen-rich environments [53]. Moore et al. [54] found that the expression level of *MMP14* increases significantly in hypoxic environments, and this is mediated by an interaction between the transcription factor HIF-1α and the *MMP14* gene promoter region. Therefore, the interaction between HIF-1α and *MMP14* may play an important role in the adaptation of Tibetan sheep to harsh, low-oxygen environments at high altitudes. *GNG2* (G protein subunit gamma 2) is a small γ2 subunit in heterotrimeric G proteins, which are involved in a variety of signaling processes and play important roles in cell proliferation, differentiation, and angiogenesis [55]. In a population genetics study of Tibetan wild boars [56], 268 genes associated with plateau adaptation were detected, among which the *GNG2* gene was strongly associated with the response to hypoxia, further suggesting that this gene is associated with adaptation to hypoxia in Tibetan sheep. Jia et al. [57] conducted a transcriptome analysis of lung tissues of Tibetan pigs at high and low altitudes and identified the potential hypoxia adaptation regulator *ATF6* (activating transcription factor 6), which is consistent with our findings. The *ATF6* gene may play a role in adaptation to low-oxygen environments in Tibetan sheep.

In low-oxygen environments, mammals can compensate for an oxygen deficiency through glycolysis pathways [58,59]. The *PGAM2* (phosphoglycerate mutase 2) gene is involved in regulating glycolysis and plays an important role in the glycolytic pathway. There is evidence that short-term exposure to low oxygen and low pressure can induce significant upregulation of glycolytic enzymes (PGAM) in the cortex of rats [60]. González-Cinca et al. [61] found that the mRNA levels and activity of PGAM increased significantly in the erythrocytes of juvenile rats after a hypoxic treatment. *PPP3R1* (protein phosphatase 3) is a regulatory subunit of protein phosphatase and plays an important role in regulating Ca^2+^ calmodulin [62]. Calcium-regulated phosphatase can maintain the structure and function of blood vessels, as well as the proliferation of vascular smooth muscle cells [21]. Wei et al. [21] identified that the *PPP3R1* gene is associated with the high-altitude adaptations of Tibetan sheep. Wang et al. [63] conducted a whole-genome analysis of copy number variants from 16 yak populations and identified various genes related to low-oxygen adaptation, including *GSTCD* (glutathione *S*-transferase C-terminal domain containing). *PPARA* (peroxisome proliferator-activated receptor alpha) is the main transcriptional regulator of fatty acid oxidase, regulating the normal metabolism of lipids [64]. It mainly participates in the HIF pathway and is regulated by the *HIF* gene. Simonson et al. [65] identified *PPARA* as a candidate gene for hypoxia adaptation, with important roles in fat metabolism. *PPARA* is regulated by feedback from HIF, and SNPs in this gene are associated with low hemoglobin levels in plateau Tibetans. In addition, Kennedy et al. [66] observed that, under hypoxic conditions, the expression of the *PPARA* gene related to fatty acid oxidation in rats was downregulated, indicating that the expression and transcriptional activity of *PPARA* is directly affected by hypoxic stimulation. Furthermore, in rats, *PPARA* is downregulated under an insufficient oxygen supply, leading to a decrease in fatty acid oxidation and an increase in the supply of carbohydrates, thereby protecting the myocardium by decreasing myocardial oxygen consumption [67]. Holden et al. [68] also verified that reduced fatty acid oxidation may confer adaptation to the hypoxic environment. Therefore, in a high-altitude, low-oxygen environment, Tibetan sheep may be protected by downregulating *PPARA* to reduce fatty acid oxidation and decrease oxygen consumption.

## 5. Conclusions

In this study, we employed whole-genome resequencing technology to detect signals of selection in OL, ZSJ, and AW, uncovering genes (*HIF1AN*, *PDGFA*, *PDGFD*, *ANXA2*, *SOCS2*, *NOXA1*, *WNT7B*, *MMP14*, *GNG2*, *ATF6*, *PGAM2*, *PPP3R1*, *GSTCD*, and *PPARA*) and pathways (the Ras, melanoma, VEGF, and PPAR signaling pathways) that are potentially related to high-altitude hypoxia adaptation in Tibetan sheep. However, the specific mechanisms of action need to be confirmed in further studies. These findings improve our understanding of genetic selection in the high-altitude adaptations of Tibetan sheep and are expected to serve as a valuable reference for future research on high-altitude livestock resources.

## Figures and Tables

**Table 1:** Information on the sheep populations in this study.

Breed	Abbr.	Sex	Age	Size	Location	Altitude	Longitude and Latitude
Oula sheep	OL	Male	Adult	20	Oula Township, Maqu County, Gannan Tibetan Autonomous Prefecture, Gansu Province	3501 m	N: 33°51′312″ E: 101°52′424″
Zashijia sheep	ZSJ	Male	Adult	20	Yocai Township, Qumalai County, Yushu Tibetan Autonomous Prefecture, Qinghai Province	4269 m	N: 34°14′866″ E: 95°80′422″
Awang sheep	AW	Male	Adult	20	Awang Township, Gongjue County, Changdu, Xizang Autonomous Region	4643 m	N: 30°12′101″ E: 98°63′98″

## Data Availability

Data are available upon request due to privacy/ethical restrictions.

## Supplementary Materials

The following supporting information can be downloaded at: https://www.mdpi.com/article/10.3390/ani14223212/s1, Table S1: GO enrichment (AW vs. OL); Table S2: GO enrichment (ZSJ vs. OL); Table S3: GO enrichment (shared genes); Table S4: KEGG enrichment (AW vs. OL); Table S5: KEGG enrichment (ZSJ vs. OL); Table S6: KEGG enrichment (shared genes).
